# Supplementary material for: Content-rich biological network constructed by mining PubMed abstracts
Source: BMC Bioinformatics. 2004 Oct 8;5:147. doi: 10.1186/1471-2105-5-147 (PMC528731; doi:10.1186/1471-2105-5-147)
Supplement: Additional File 2 — The original results of the above study (non-essential files are deleted to keep the file size under the limit set by BMC bioinformatics). [file 1471-2105-5-147-S2.bz2 › chilibotAdditionalFile2/dip10/389ID11297552E9864/html/PDGFRB_PDGFB.html]

 


 **PDGFRB** and **PDGFB** 
  
Found 434 abstracts in PubMed, retrieved 10.  
 

 What does Google say? 
 PDF only 
| .edu only 

---

**Interactive relationship** (e.g. stimulation, inhibition, etc)

**Non-interactive relationship** (e.g. studied together, co-existance, homology, etc.)

- We previously reported that the platelet derived growth factor B chain  [ **PDGFB** ]  PDGF B PDGF receptor PDGFR  [ **PDGFRB** ]  axis is involved in tubular regeneration after ischemia reperfusion injury of the kidney.  Ref: 12819032 Am J Pathol, 2003
- Immunohistochemical procedures were performed to detect the following protein antigens CD99, interleukin IL 1alpha, IL 6, transforming growth factor TGF alpha, conventional c protein kinase C cPKC alpha, cPKC betaII, phosphorylated p PKC alpha betaII, c kit CD117, platelet derived growth factor  [ **PDGFB** ]  receptor PDGFR  [ **PDGFRB** ]  alpha, PDGFR  [ **PDGFRB** ]  beta, epidermal growth factor receptor EGFR, human epidermal growth factor receptor HER 2 neu, cathepsin D, angiotensin converting enzyme ACE, angiotensin II type 1 AT1 receptor, p21ras, the alpha subunit of farnesyl and geranylgeranyl transferase FTalpha GGTalpha, phospho p c Jun N terminal kinase p JNK, p p38 mitogen activated protein kinase MAPK, cyclin D1, c Jun, Ki 67, bcl 2, TGF beta1 latency associated peptide LAP, TGF betaRII, and cyclooxygenase COX 2.  Ref: 12817616 Ann Clin Lab Sci< MedlineTA>Ann Clin Lab Sci, 2003
- Imatinib Gleevec formerly STI571 is an orally bioavailable rationally developed inhibitor of the tyrosine kinases Bcr Abl, Kit, and platelet derived growth factor  [ **PDGFB** ]  receptor PDGFR  [ **PDGFRB** ] .  Ref: 12783370 Semin Hematol, 2003
- cKit and platelet derived growth factor  [ **PDGFB** ]  receptor PDGFR  [ **PDGFRB** ]  are receptor tyrosine kinases expressed in the testis, are involved in testosterone production, and are inhibited by imatinib.  Ref: 12801741 Lancet, 2003
- OBJECTIVE To study the cleavage activity of hammerhead ribozyme targeting at platelet derived growth factor  [ **PDGFB** ]  receptor beta  [ **PDGFRB** ]  subunit PDGFR  [ **PDGFRB** ]  beta mRNA in hepatic stellate cells HSCs and its effect on the biological characters of HSCs.  Ref: 12773241 Zhonghua Gan Zang Bing Za Zhi, 2003
- Recently, tumors with platelet derived growth factor  [ **PDGFB** ]  receptor PDGFR  [ **PDGFRB** ]  a mutations have been described in GISTs with wild type KIT.  Ref: 12781070 Curr Oncol Rep, 2003
- Sodium hydrogen exchanger regulatory factor 1 and 2 NHERF 1 and NHERF 2 are adaptor proteins that regulate renal electrolyte transport and interact with the platelet derived growth factor  [ **PDGFB** ]  receptors PDGFR  [ **PDGFRB** ] .  Ref: 12865627 PathobiologyPathobiology, 2003
- Because activation of platelet derived growth factor  [ **PDGFB** ]  PDGF receptor tyrosine kinase PDGFr  [ **PDGFRB** ]  TK influences these processes and promotes restenosis, TKI963, an inhibitor of the PDGFr  [ **PDGFRB** ]  TK was discovered, and its efficacy was evaluated in blocking stent induced restenosis as analyzed by intravascular ultrasound IVUS.  Ref: 12775958 J Cardiovasc Pharmacol, 2003
- Effect of ribozyme against platelet derived growth factor  [ **PDGFB** ]  receptor beta  [ **PDGFRB** ]  subunit mRNA on the biological characters of hepatic stellate cells < ArticleTitle>.  Ref: 12773241 Zhonghua Gan Zang Bing Za Zhi, 2003
- A possible regulation of this transport system by platelet derived growth factor  [ **PDGFB** ]  receptor PDGFr  [ **PDGFRB** ]  with tyrosine kinase activity is also demonstrated.  Ref: 12822894 IUBMB Life, 2003
